# Prevalence of nocturia among community-dwelling adults: a population-based study in Malaysia

**DOI:** 10.1186/s12894-021-00860-1

**Published:** 2021-06-29

**Authors:** Hui-Yin Yow, John Jeh Lung Tiong, Chun-Wai Mai, Esther van der Werf, Zulkifli Md Zainuddin, Charng-Chee Toh, Kay-Seong Ngoo, Eng-Hong Goh, Ahmad Nazran Fadzli, Sze-Han Lok, Teng-Aik Ong

**Affiliations:** 1 School of Pharmacy, Faculty of Health and Medical Sciences, Taylor’s University (Lakeside Campus), Subang Jaya, Malaysia; 2 Ferring Pharmaceuticals Malaysia & Singapore, Kuala Lumpur, Malaysia; 3 School of Pharmacy, International Medical University, Kuala Lumpur, Malaysia; 4 Louis Bolk Institute, Bunnik, The Netherlands; 5 Urology Unit, Department of Surgery, Universiti Kebangsaan Malaysia, Kuala Lumpur, Malaysia; 6 Department of Urology, Hospital Selayang, Selangor, Malaysia; 7 Department of Surgery, Hospital Angkatan Tentera Tuanku Mizan, Kuala Lumpur, Malaysia; 8 Urology, Nephrology & Men’s Health Clinic, Prince Court Medical Centre, Kuala Lumpur, Malaysia; 9 Department of Surgery, Faculty of Medicine, University of Malaya, 50603 Kuala Lumpur, Malaysia

**Keywords:** Prevalence, Nocturia, Awareness, Malaysia, Survey

## Abstract

**Background:**

Nocturia is widely prevalent condition with detrimental effects on quality of life and general health. In Malaysia, there is a lack of up-to-date prevalence study on nocturia. This study aimed to investigate the prevalence of nocturia and awareness pertaining to nocturia among Malaysian adults.

**Methods:**

A cross-sectional population-based study was conducted among Malaysian adults aged ≥ 18 years old. The data was collected by mixed mode self-administered questionnaire from May 2019 to September 2019. Nocturia was defined as one or more voids at night.

**Results:**

There were a total of 4616 respondents with 74.5% of response rate. The overall prevalence of nocturia among Malaysian adults was found to be 57.3%. In multivariate analysis, respondents aged 31–40 (1.91 [1.52–2.40]) or > 60 years old (2.03 [1.48–2.71]), and those who presented with hypertension (2.84 [2.28–3.53]), diabetes mellitus (1.78 [1.42–2.25]), renal disease (3.58 [1.93–6.63]) or overactive bladder (1.61 [1.10–2.35]) were associated with higher prevalence of nocturia. A significantly lower disease prevalence (*p* < 0.05) was noted among those aged 41–50 (0.73 [0.59–0.91]), male (0.78 [0.69–0.88]) and Chinese (0.47 [0.30–0.74]) or Indian (0.34 [0.21–0.54]) ethnicities. A total of 37.3% of respondents with nocturia reported that they faced sleeping difficulty about half the time or more after waking up in the middle of night. Those who had ≥ 2 voids per night experienced significantly higher mean bother score than those who had 1 void per night (*p* < 0.001). Approximately half (56.7%) of all respondents were not aware that night time urination is a medical condition. Only 25.2% of respondents with nocturia had sought medical attention for their nocturia.

**Conclusions:**

The prevalence of nocturia among Malaysian adults is high and strongly influenced by age, sex, race and comorbidities. However, the general awareness pertaining to nocturia being a health issue remains low among Malaysians. The findings also highlighted the impact of nocturia on sleep and the need for nocturia education to better address this disease.

**Supplementary Information:**

The online version contains supplementary material available at 10.1186/s12894-021-00860-1.

## Background

Nocturia is defined as ‘the complaint that the individual has to wake at night one or more times to void’ by International Continence Society (ICS) [1]. Until recently, the definition has been revised as ‘waking up to pass urine during the main sleep period’ [2]. Nocturia can affect all age groups although the disease prevalence does increase with age. Bosch and Weiss reviewed the nocturia prevalence studies in community based, predominantly populations in the western countries and some Asian countries (including China, Taiwan, Japan, Korea, Hong Kong and Singapore) [3]. They reported that prevalence of nocturia among younger populations (20–40 years old) with one or more voids per night varies from 11–44% whilst those with two or more voids per night has been reported to range between 2–18% [3]. Nevertheless, nocturia is more frequent among the older populations (> 70 years old), with 69–93% having one or more voids per night while 28–62% reported two or more voids per night [3]. Similarly, a longitudinal study conducted by van Doorn and colleagues in the Dutch municipality Krimpen aan den IJssel showed that the prevalence of nocturia increases over time and with increasing age among men aged 50 to 78 years [4].

Several studies have demonstrated that nocturia has negative impact on quality of life and general health [5–8]. Increase in the number of voids per night has serious consequences on sleep, symptoms, morbidity from fatigue and falls and health-related quality of life [5–7]. Unfortunately, nocturia is often unreported, with the general lack of knowledge that nocturia is a treatable medical condition being cited as a crucial barrier to treatment seeking [9].

In Malaysia, there is lack of up-to-date prevalence studies on nocturia, only very limited studies are available as reference. A cross-sectional study had been carried out in Institute of Urology and Nephrology (IUN), Kuala Lumpur, Malaysia in 2007 and reported that the prevalence of nocturia was 29% among patients presented with lower urinary tract symptoms (LUTS) [10]. Similarly, Low and colleagues reported that high prevalence (19%) of LUTS was found among women in Northern Malaysia in 2006 and many of them did not seek treatment due to low disease awareness [11]. Most of these studies were at least 10 years ago, and thus the relevance remains questionable. Therefore, the aim of this present population-based study was to investigate the prevalence of nocturia and awareness pertaining to nocturia among community-dwelling adults in Malaysia.

## Methods

**Fig. 1:**
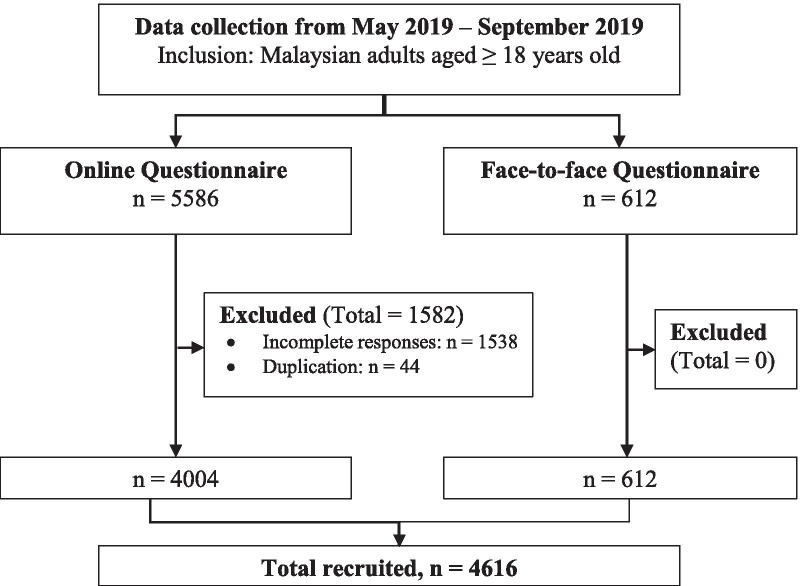
Study protocol of subject inclusion and exclusion

A cross-sectional population-based study was conducted among community-dwelling Malaysian adults aged ≥ 18 years old across all states in Malaysia from May 2019 to September 2019 (Fig. 1). Convenience sampling was applied to obtain responses from any Malaysian adult aged ≥ 18 years old. The respondents were approached based on the age population percentage reported by Department of Statistics Malaysia (https://www.dosm.gov.my). Individuals who were unable to read or understand English, Malay or Chinese, or with cognitive impairment or active mental illness, and all incomplete responses were excluded. A complete response was considered when the respondent answered all required questions until the last page of the survey, whereas for the questionnaire that was not done until the last page of survey was categorised as ‘incomplete response’.

### Questionnaire

The questionnaire consisted of three sections was developed for this study (Additional file 1). The first section of the research questionnaire consisted of items to extract the demographic data of the subjects. The second part was made up of three domains, namely present medical condition and disease pattern (2 items), night urination and sleeping issues (5 items) and treatment-seeking behaviour (2 items). Nocturia was defined as one or more voids at night based on the definition from ICS [1]. Those who had to wake up from sleep at night to urinate at least once were required to answer all items, while those without night urination were exempted from some questions pertaining to night urination and sleeping issues and treatment-seeking behaviour. The questionnaire was validated by a panel of experts on content validity and clarity. The English version of the questionnaire was checked by a native English speaker. The questionnaire was translated into Malay and Chinese languages based on ISPOR guideline with two forward-translation and two back-translation [12]. Three languages of questionnaire were pre-tested with a pilot study (n = 40, respectively) to confirm on the simplicity of language used and to assess the comprehension of the questions. Feedbacks were taken into consideration to incorporate into the final questionnaire. A second pilot study was conducted after the changes made to the questions, followed by a retest of the pilot study to assess internal consistency.

### Data collection

Previous to the data collection, the researchers or the trained research assistants used a pre-trained structured protocol to introduce the instrument to the participants based on the inclusion criteria. In order to increase the response rate and outreach nationwide, data was collected by mixed mode, i.e. face-to-face and online modes. Face-to-face data collection was important to address the low level of technology literacy and/or limited access to the internet among certain segments of the population i.e. the elderly as well as the suburban and rural folks. It took place in public areas i.e. shopping malls, parks, restaurants, cafeteria and markets in rural and urban areas. The approached subjects spent approximately 5–10 min on the printed self-administered questionnaires. For the online mode, a third-party online platform was utilised to collect the responses. An information sheet about this research study along with a written consent form were attached to each copy of the questionnaire. All subjects signed the written informed consent form prior to answering the questionnaire.

### Data analysis

Continuous variables were presented as mean and standard deviation; whilst categorical variables were presented as frequency and percentage. All data collected were tabulated and analysed using the Statistical Package for Social Sciences software, version 24.0. Pearson’s chi-squared (χ^2^) test was used to assess the significance in prevalence differences between dichotomous variables, such as sex, age group and employment status (or Fisher’s exact test when the frequency of respondents was less than 5 for any category). For ordinal or continuous variables (e.g. age and degree of bother), the Mann–Whitney U test was used to determine the difference between two independent groups (or Kruskal Wallis test if there was more than two independent groups). Univariable analysis were performed for the respondents’ characteristics in relation to prevalence of nocturia using logistic regression analysis. Variables with an association of *p* < 0.05 were selected for multivariable analysis using logistic regression analysis. Significance was defined as *p* < 0.05.

## Results

The response rate was 74.5%, meaning that a total of 6198 respondents across the states in Malaysia participated in this study. There were 1538 of incomplete responses and 44 duplication of responses, which were excluded from the study. The demographic data of the study population was shown in Table 1. The comparison of demographic characteristics of study population with and without nocturia was shown in Table 2. Statistical analysis revealed that there was significant difference in prevalence between mean age, sex, race employment status and comorbidities (*p* < 0.05, respectively).

**Table 1:** Demographic characteristic of study population

Variable	Overall (n, %)	Mode of questionnaire		*p* value*
		Online (n, %)	Face-to-Face (n, %)	
Total respondents	4616 (100)	4004 (86.7)	612 (13.3)	
Mean age (years ± SD)	38.4 ± 14.58	38.0 ± 14.08	40.9 ± 17.31	0.025^φ^
18–30	1794 (38.9)	1573 (39.2)	221 (36.2)	
31–40	889 (19.3)	835 (20.9)	54 (8.8)	
41–50	760 (16.5)	692 (17.3)	68 (11.1)	
51–60	865 (18.7)	664 (16.6)	201 (32.9)	
> 60	307 (6.7)	240 (6.0)	67 (11.0)	
Sex				
Male	1982 (42.9)	1646 (41.1)	336 (54.9)	< 0.001^#^
Female	2634 (57.1)	2358 (58.9)	276 (45.1)	
Race				
Malay	1796 (38.9)	1222 (30.5)	574 (94.1)	< 0.001^#^
Chinese	2126 (46.1)	2115 (52.8)	11 (1.8)	
Indian	595 (12.9)	574 (14.4)	21 (3.4)	
Others	96 (2.1)	92 (2.3)	4 (0.7)	
Employment status				
Student	920 (19.9)	724 (18.1)	196 (32.0)	< 0.001^#^
Employed for wages	2659 (57.4)	2370 (59.2)	280 (45.8)	
Self-employed	394 (8.5)	319 (8.0)	75 (12.2)	
Unemployed	652 (14.1)	591 (14.7)	61 (10.0)	
Comorbidities				
Hypertension	638 (13.8)	502 (12.5)	136 (22.2)	< 0.001^#^
Diabetes mellitus	524 (11.4)	402 (10.0)	122 (19.9)	< 0.001^#^
Renal disease	90 (1.9)	39 (1.0)	51 (8.3)	< 0.001^#^
Heart failure	22 (0.5)	16 (0.4)	6 (1.0)	0.052^#^
Enlarged prostate	61 (1.3)	59 (1.5)	2 (0.3)	0.021^#^
Overactive bladder	141 (3.1)	121 (3.0)	20 (3.3)	0.742^#^
Prostate cancer	9 (0.2)	8 (0.2)	1 (0.2)	1.000^ϕ^
Bladder cancer	6 (0.1)	6 (0.1)	0 (0)	1.000^ϕ^
Snoring	781 (16.9)	714 (91.4)	67 (11)	< 0.001^#^
Prevalence of Nocturia	2646 (57.3)	2166 (54.1)	480 (78.4)	< 0.001^#^

*The statistical significance level was 0.05; ^φ^Mann–Whitney U test; ^#^Chi-square test; ^ϕ^Fisher’s Exact test; SD: standard deviation; n = 4616

**Table 2:** Demographic characteristic of study population with and without nocturia

Variable	Nocturia (n, %)	No nocturia (n, %)	*p* value*
Total respondents	2646 (57.3)	1970 (42.7)	
Mean age (years ± SD)	39.14 ± 14.98	37.33 ± 13.96	< 0.001^φ^
18–30	948 (35.8)	846 (43.0)	
31–40	591 (22.4)	298 (15.1)	
41–50	366 (13.8)	394 (20.0)	
51–60	508 (19.2)	357 (18.1)	
> 60	233 (8.8)	74 (3.8)	
Sex			
Male	1103 (41.7)	879 (44.6)	0.046^#^
Female	1543 (58.3)	1091 (55.4)	
Race			
Malay	1239 (46.9)	557 (28.2)	< 0.001^#^
Chinese	1087 (41.1)	1039 (52.8)	
Indian	253 (9.6)	342 (17.4)	
Others	65 (2.4)	31 (1.6)	
Employment status			
Student	490 (18.5)	430 (21.8)	0.019^#^
Employed for wages	1555 (58.8)	1095 (55.6)	
Self-employed	216 (8.2)	178 (9.0)	
Unemployed	385 (14.5)	267 (13.6)	
Comorbidities			
Hypertension	500 (18.90)	138 (7.0)	< 0.001^#^
Diabetes mellitus	369 (13.95)	155 (7.9)	< 0.001^#^
Renal disease	77 (2.91)	13 (0.7)	< 0.001^#^
Heart failure	17 (0.64)	5 (0.3)	0.058^#^
Enlarged prostate	41 (1.55)	20 (1.0)	0.116^#^
Overactive bladder	93 (3.51)	48 (2.4)	0.035^#^
Prostate cancer	6 (0.23)	3 (0.2)	0.741^ϕ^
Bladder cancer	4 (0.15)	2 (0.1)	1.000^ϕ^
Snoring	471 (17.80)	310 (15.7)	0.064^#^

*The statistical significance level was 0.05; ^φ^Mann-Whitney U test; ^#^Chi-square test; ^ϕ^Fisher’s Exact test; SD: standard deviation; n = 4616

**Fig. 2:**
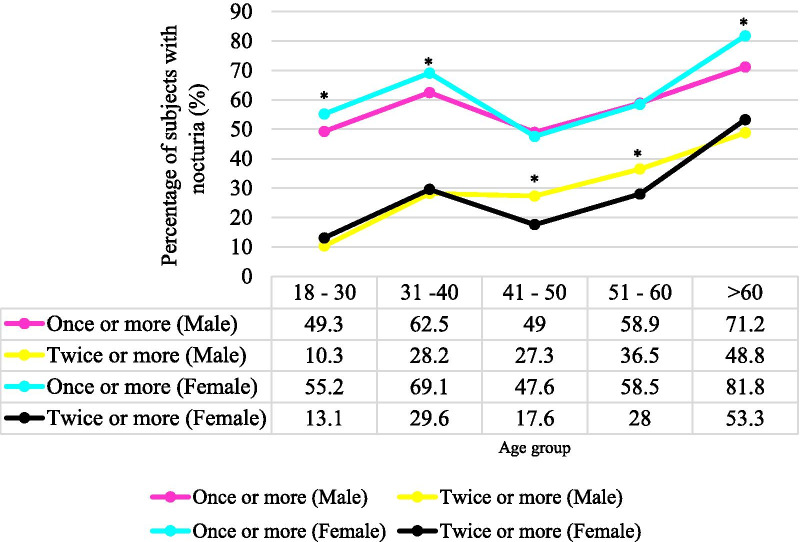
The prevalence of nocturia based on sex and frequency of nocturia. n = 4616. *According to the Chi-Square test, younger (18–40 years old) and older women (> 60 years old) were more commonly to have one void per night than men from the same age group (*p* < 0.05). Whereas middle-aged men (41–60 years old) were more likely to have two voids per night as compared to the middle-aged women (*p* < 0.05)

The overall prevalence of nocturia (at least one void per night) among Malaysian adults was 57.3% (Table 2), with 34.1%, 15.3% and 7.9% having an of an average of one void per night, two voids per night and three or more voids per night respectively. Figure 2 illustrated the increasing trend of prevalence with age in both sex and frequency of nocturnal voids was associated significantly with sex in different age groups. Younger (18–40 years old) and older women (> 60 years old) were more likely to have one void per night than men from the same age group (*p* < 0.05). Whereas middle-aged men (41–60 years old) were more likely to have two voids per night as compared to the middle-aged women (*p* < 0.05; Fig. 2).

**Fig. 3:**
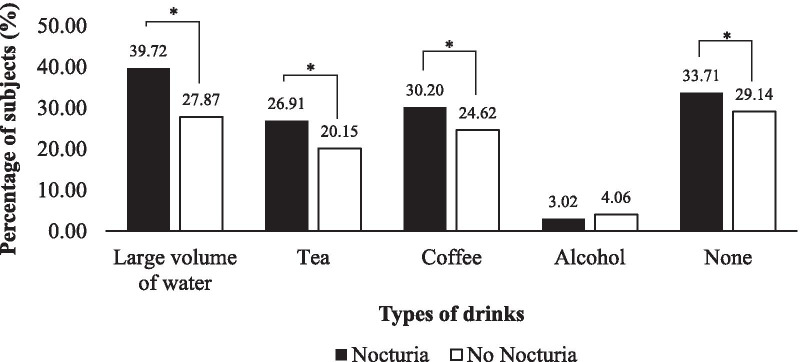
Relationship between prevalence of nocturia and drinks consumption in the late evening. n = 4616. *According to the Chi-Square test, the prevalence of nocturia was significantly associated with the drink consumption in the late evening, including large volume of water, tea and coffee (*p* < 0.001). The prevalence of nocturia was significant among those without any drink consumption in the late evening (*p* < 0.001)

The variables univariately associated with nocturia are presented in Table 3. Age group, sex, race, employments status and comorbidities such as hypertension, diabetes mellitus, renal disease and overactive bladder were significantly associated with the prevalence of nocturia. In the multivariate analysis, age group between 31 and 40 or > 60 years old, and those who were presented with comorbidities included hypertension, diabetes mellitus, renal disease or overactive bladder were associated with significantly higher prevalence of nocturia, whereas age group of 41–50, male and Chinese or Indian were associated with a decreased prevalence (Table 3). This study also revealed that the prevalence of nocturia was significantly increased with the drink consumption in the late evening, including large volume of water, tea and coffee (Fig. 3; *p* < 0.001) although evening drinking habit was only one of the few factors predisposing to nocturia since the prevalence of nocturia was also significantly high among those without any drink consumption in the late evening (*p* < 0.001).

**Table 3:** Univariable and multivariable analysis for respondents’ characteristic in relation to prevalence of nocturia

	Univariate Analysis*		Multivariate Analysis*^,#^	
	OR (95% CI)	*p* value	OR (95% CI)	*p* value
Age group				
18–30	0.741 (0.658–0.835)	< 0.001	1.161 (0.931–1.449)	0.185
31–40	1.613 (1.383–1.880)	< 0.001	1.909 (1.521–2.396)	< 0.001
41–50	0.642 (0.549–0.750)	< 0.001	0.733 (0.592–0.908)	0.005
51–60	1.073 (0.923–1.803)	0.358	Not included	–
> 60	2.473 (1.891–3.234)	< 0.001	2.027 (1.478–2.708)	< 0.001
Sex				
Male	0.887 (0.789–0.998)	0.046	0.779 (0.687–0.884)	< 0.001
Female	1.127 (1.002–1.268)	0.046	–	–
Race				
Malay	2.235 (1.974–2.531)	< 0.001	0.891 (0.569–1.396)	0.615
Chinese	0.625 (0.556–0.703)	< 0.001	0.471 (0.302–0.735)	0.001
Indian	0.503 (0.423–0.599)	< 0.001	0.336 (0.210–0.537)	< 0.001
Others	1.576 (1.023–2.427)	0.039	–	
Employment status				
Student	0.814 (0.704–0.941)	0.005	1.154 (0.911–1.463)	0.235
Employed for wages	1.139 (1.012–1.281)	0.031	1.141 (0.963–1.352)	0.129
Self-employed	0.895 (0.727–1.101)	0.294	Not included	–
Unemployed	1.086 (0.918–1.285)	0.336	Not included	–
Comorbidities				
Hypertension	3.093 (2.536–3.772)	< 0.001	2.837 (2.281–3.530)	< 0.001
Diabetes mellitus	1.898 (1.558–2.312)	< 0.001	1.783 (1.416–2.245)	< 0.001
Renal disease	4.512 (2.500–8.145)	< 0.001	3.579 (1.933–6.625)	< 0.001
Heart failure	2.541 (0.936–6.900)	0.067	Not included	–
Enlarged prostate	1.535 (0.896–2.627)	0.119	Not included	–
Overactive bladder	1.459 (1.024–2.077)	0.036	1.606 (1.097–2.352)	0.015
Prostate cancer	1.490 (0.372–5.966)	0.573	Not included	–
Bladder cancer	1.490 (0.273–8.142)	0.645	Not included	–
Snoring	1.160 (0.991–1.357)	0.064	Not included	–

*Based on logistic regression analysis, the statistical significance level was 0.05; ^#^Variables with an association of *p* < 0.05 were selected for a multivariable analysis; OR: Odd ratio; CI: Confidence interval; n = 4616

**Fig. 4:**
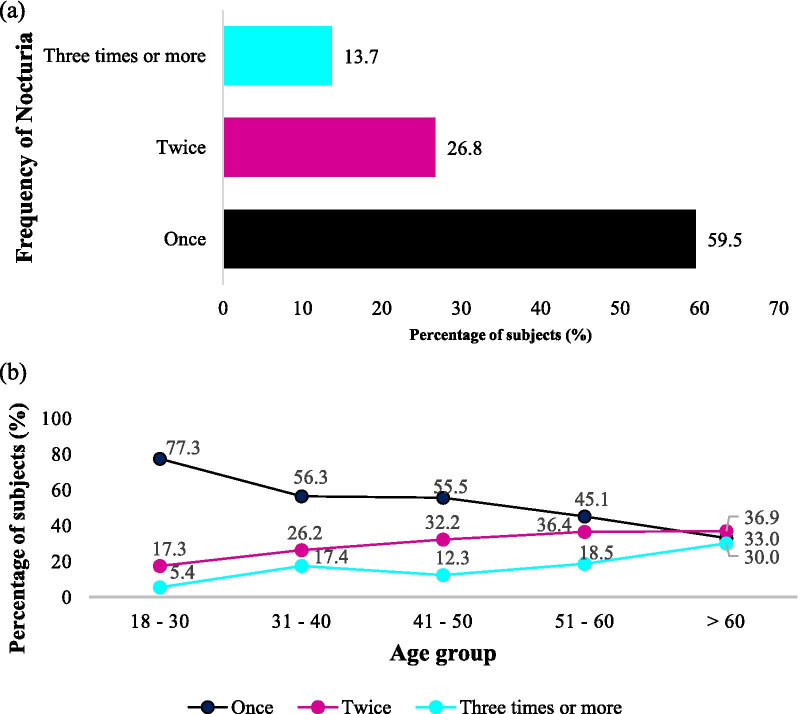
Frequency of nocturia **a** among respondents with nocturia, **b** in different age groups. n = 2646

From the sub analyses, the frequency of nocturnal voids among respondents with nocturia and among different age groups has been depicted in Fig. 4. Those who were more than 60 years old were more likely to experience more than one nocturnal void as compared to their younger counterparts (Fig. 4). The mean age for those with once, twice and three or more times of nocturnal voids was 35.51 ± 14.03, 43.41 ± 14.49 and 46.55 ± 14.97, respectively. The association was significant, which indicated that frequency of nocturnal void is higher in older age groups (*p* < 0.001).

The disease pattern of nocturia encountered by the respondents with nocturia has been summarised in Table 4. Post urination dribble was the only symptom that differed significantly in male respondents with nocturia compared to female (*p* < 0.001). Those with higher age (> 50 years old) were more likely to present with almost all the symptoms (*p* < 0.05).

**Table 4:** Disease pattern of nocturia among study population based on sex and age

Symptom	Sex (n, %)		Age group (n, %)	
	Male	Female	18–50	> 50
Total respondents	1103 (41.7)	1543 (58.3)	1905 (72.0)	741 (28.0)
Post urination dribble	172 (15.6)	139 (9.0)	237 (12.4)	74 (10.0)
	*p* < 0.001*		*p* = 0.078*	
Frequent urination during the day	276 (25.0)	274 (17.8)	438 (23.0)	212 (28.6)
	*p* = 0.644*		*p* = 0.003*	
Frequent urinary urgency	129 (11.7)	168 (10.9)	155 (8.1)	142 (19.2)
	*p* = 0.516*		*p* < 0.001*	
Urinary Incontinence	90 (8.2)	124 (8.0)	73 (3.8)	141 (19.0)
	*p* = 0.909*		*p* < 0.001*	
Slow urine flow	63 (5.7)	78 (5.1)	69 (3.6)	72 (9.7)
	*p* = 0.458*		*p* < 0.001*	
Feeling of incomplete bladder emptying after urination	143 (13.0)	165 (10.7)	166 (8.7)	142 (19.8)
	*p* = 0.072*		*p* < 0.001*	

*Based on Chi-square test, the statistical significance level was 0.05; n = 2646

In terms of impact of nocturia on sleep disturbance, 37.3% of respondents with nocturia reported that they faced sleeping difficulty about half the time or more after waking up in the middle of night, particularly among those with ≥ 2 voids per night (66.5%), aged > 50 (40.6%) and employed for wages (62.3%) (Table 5; *p* < 0.001). As consequences of sleep disturbance due to nocturia, they complaint of fatigue (60.9%), difficulty in concentrating (43.2%), bad mood (41.2%), lack of motivation to work (34.5%), forgetfulness (24.6%), depression (15.6%), lack of sex drive (4.61%) and erectile dysfunction (3.5%; only male respondents). On a scale of 1 to 10 for degree of bother, those who had two voids as well as ≥ 3 voids per night experienced significantly higher mean bother score (3.96 ± 2.41 and 3.91 ± 2.20, respectively) than those who had one void per night (3.29 ± 2.63) (*p* < 0.001).

**Table 5:** The impact of nocturia on sleep disturbance and factors affecting the sleep disturbance

Variable	Degree of sleep disturbance (n, %)		*p* value*
	Less than half the time	About half the time or more	
Total respondents	1655 (62.7)	983 (37.3)	
Frequency of nocturia			
Once	1241 (75.0)	329 (33.5)	< 0.001
Twice	273 (16.5)	433 (44.0)	
Three times or more	141 (8.5)	221 (22.5)	
Age			
18–30	730 (44.1)	215 (21.9)	< 0.001
31–40	375 (22.6)	215 (21.9)	
41–50	210 (12.7)	154 (15.6)	
51–60	233 (14.1)	273 (27.8)	
> 60	107 (6.5)	126 (12.8)	
Sex			
Male	681 (41.1)	417 (42.4)	0.521
Female	974 (58.9)	566 (57.6)	
Employment status			
Student	391 (23.6)	98 (10.0)	< 0.001
Employed for wages	938 (56.7)	613 (62.3)	
Self-employed	116 (7.0)	98 (10.0)	
Unemployed	210 (12.7)	174 (17.7)	

*Based on Chi-square test, the statistical significance level was 0.05; n = 2638

**Fig. 5:**
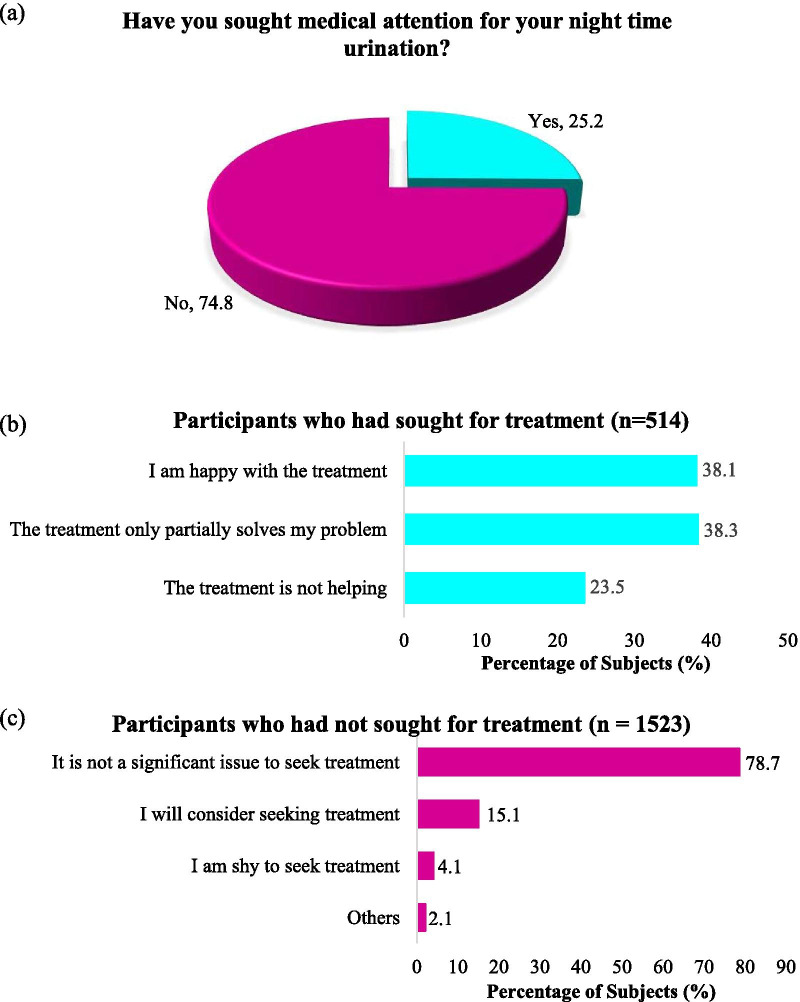
Treatment seeking behaviour among participants with nocturia. n = 2037. **a** The overall treatment seeking behaviour among participants with nocturia. **b** The responses from those participants who had sought for treatment. **c** The responses from participants who had not sought for treatments

Our study also found that the awareness on nocturia was poor among study population. Out of 4616 respondents, 56.7% of them were not aware that night time urination is a medical condition. Unfortunately, the majority (62.2%) of respondents with nocturia did not think that it is a medical condition. Only a minority (25.2%) of respondents with nocturia had sought medical attention for their nocturia (Fig. 5). There was a correlation between treatment-seeking behaviour with age, sex, employment status, frequency of nocturia, degree of sleep disturbance, bother score and nocturia disease awareness among respondents with nocturia (*p* < 0.05; Table 6). A total of 60.7% out of 4616 respondents had sought or would consider seeking medical attention for nocturia from urologist. There were 39.6% and 21.3% who opted for general practitioner and gynecologist, respectively. This was followed by 17.7% and 17.2% who had chosen pharmacist and nephrologist, respectively.

**Table 6:** Characteristics comparison between respondents who had and did not seek treatment

Variable	Treatment seeking behaviour (%)		*p* value*
	Yes (n = 514)	No (n = 1523)	
Age (years ± SD)	44.43 ± 16.34	38.59 ± 14.67	< 0.001^φ^
Sex			
Male	49.4	42.9	0.011^#^
Female	50.6	57.1	
Employment status			
Student	14.6	19.7	< 0.001^#^
Employed for wages	51.6	61.5	
Self-employed	10.7	7.3	
Unemployed	23.2	11.4	
Frequency of nocturia			
Once	47.3	62.1	< 0.001^#^
Twice	34.2	27.5	
Three times or more	18.5	10.4	
Degree of sleep disturbance			
Less than half the time	49.0	62.1	< 0.001^#^
About half the time or more	51.0	37.9	
Bother score (mean ± SD)	4.51 ± 2.55	3.19 ± 2.4	< 0.001^φ^
Awareness of nocturia as a medical condition			
Yes	54.3	35.4	< 0.001^#^
No	45.7	64.6	

*The statistical significance level was 0.05; ^φ^Mann-Whitney U test; ^#^Chi-square test; SD: standard deviation; n = 2037

## Discusssion

This study presented the first nation-wide report on prevalence of nocturia (at least one void at night) in a multiracial population of Malaysia. The overall prevalence of nocturia among Malaysian adults was high (57%), which was comparable with those reported from studies in Asian population, yet relatively higher compared to Western populations. Liew and colleagues reported that the prevalence of nocturia among Singaporean adults was 55% [13]. Meanwhile, the reported prevalence were 46% in the Dutch population [14], 31% in the US population [5] and 28.4% in the Turkish population [15]. In the present study, there was a significant higher prevalence among women. This finding is consistent with the report from previous studies [13, 14, 16, 17]. Nevertheless, information on the exact etiology pertaining to high prevalence of nocturia in women is lacking. Nocturia was found to be more common among younger women (18–40 years old). This is congruent with the finding from study in Finland [17].

In addition, the prevalence of nocturia was found to increase with age, which corroborated the findings of other studies [4, 5, 18]. This may be due to various pathophysiological mechanisms that related to ageing. In the elderly, nocturia can be resulted from decline in functional bladder capacity with high post void residual, deregulation of arginine vasopressin level, sleep disorders and comorbidities including diabetes mellitus and diabetes insipidus [19]. This also further explains the finding in current study that the frequency of nocturnal voids was higher among the elderly population than younger population. On the other hand, there was higher nocturia prevalence among those 31–40 years old than those between 41 and 50 years old. The underlying etiology for this finding is unknown, but it may be due to the alcoholic beverage consumption among this age group. Based on the previous National Health and Morbidity Survey 2011 in Malaysia, those 30–39 years old presented with highest prevalence of alcohol consumption than other age groups and the drinking pattern waned by older population [20]. Nevertheless, further investigation is required to reveal the exact etiology of this finding.

Previous studies have reported the differences in prevalence of nocturia among races in the US population. The prevalence of nocturia was reported to be higher among Black and Hispanic respondents compared to the White respondents [21, 22]. Consistently, similar observation was reported among the multi-ethnic groups in Malaysia. Malay respondents had a significant higher prevalence compared to other races as revealed by the univariate analysis, yet this prevalence was not significant in multivariate analysis. Chinese and Indian respondents on the other hand, had significantly lower prevalence compared to Malay in both univariate and multivariate analyses. However, Liew and colleagues reported that there was no significant difference between Malay, Chinese and Indian respondents in Singapore [13]. Kupelian and colleagues reported that socioeconomic status was accounted for the ethnic discrepancies in prevalence of nocturia [22]. Further investigation is required in order to identify the underlying etiology of ethnic discrepancies among Malaysians in term of prevalence of nocturia.

Nocturia is strongly associated with various risk factors and comorbidities, including cardiovascular diseases, diabetes, sleep disorders, somatic and mental health [6, 13, 18, 23, 24]. This supports the findings of this study which also showed the significant higher disease prevalence among respondents with comorbidities such as hypertension, diabetes mellitus, renal disease or overactive bladder. The pathophysiological changes in hypertensive patients appear to be a significant risk factor for nocturia [25, 26]. A prolonged hyperglycemic state in diabetes mellitus patients on the other hand, may cause an alteration in urinary bladder activity, predisposing to diabetic cystopathy [27]. Diabetic cystopathy is primarily induced by paralysis of detrusor muscle and hyperglycemia-induced polyuria that can further lead to bladder hypertrophy in patients with diabetes mellitus [27]. Moreover, microvascular complications of diabetes, particularly diabetic nephropathy and neuropathy have been implicated in nocturia [28]. Diabetic nephropathy is associated with polyuria and an increase in mean void volume, whereas, diabetic neuropathy may lead to impaired bladder sensation, poor detrusor contractility and increased post void residual [28]. This explains the mechanistic link between diabetes mellitus and nocturia.

Several studies have reported that nocturia is associated with chronic kidney disease [13, 29, 30]. This may be attributed to the failure of kidneys to concentrate the urine as the renal function worsens, and ultimately leads to nocturnal polyuria and increased nocturnal urine volume in patients with chronic kidney disease [31]. Additionally, overactive bladder was also found to be one of the risk factors in this study. Indeed, nocturia is a common urinary symptom in overactive bladder patients [32]. However, in this study, there was no significant association between nocturia and benign prostatic hyperplasia, prostate cancer, bladder cancer and snoring, which had been reported in previous studies [22, 23, 33]. This could be due to the comorbidities determined by self-reporting of respondents in the present study, which might have led to underestimation of the prevalence of these comorbidities in Malaysia.

Excessive evening intake of fluid, caffeinated drinks or alcohol is known to be one of the causes of nocturnal polyuria [1]. In the current study, approximately one third of the respondents with nocturia have the habit of consuming large volume of water, tea or coffee in the late evening. This may suggest that limiting fluid or caffeinated drink intake in the late evening among Malaysian could be one of the modifiable lifestyle risk factors for nocturia. Shiri and colleagues reported that there was no significant association between coffee consumption and nocturia among 50 to 70 years old men in Finland, yet the risk of moderate or severe nocturia was twofold higher among men who drank 6 cups coffee or more per day than non-drinkers or those who consumed one cup per day for this age group [34]. Interestingly, the prevalence of nocturia was about one third of respondent despite any drink consumption. The actual prevalence of nocturia with underlying pathological causes in the population may be reflected by eliminating the potential confounding factor of drink consumption in the late evening.

LUTS can be classified according to voiding or obstructive symptoms (such as hesitancy, poor stream, straining, prolonged micturition, feeling of incomplete bladder emptying, post-micturition dribble) and storage or irritative symptoms (such as frequency, urgency, urge incontinence and nocturia). In the present study, the disease pattern of nocturia was not sex-specific, except for post-micturition dribble. This interpretation is supported by the fact that post-micturition dribble is more common in men [35]. Studies also showed that prevalence of LUTS was comparable between men and women [36, 37]. In addition, with advancing age, the prevalence and severity of LUTS increase regardless of sex [36]. This is supported by the finding of this study where elderly respondents are more likely to exhibit almost all the LUTS compared to the younger population.

Nocturia has been regarded as not only ‘bothersome’, but also negatively affects sleep and quality of life [5–8]. This study also revealed that nocturia was regarded as moderately bothersome for Malaysians, particularly those with two or more nocturnal voids. This alluded to the clinical relevance of at least 2 voids being the threshold to patient-reported impaired health-related quality of life [38]. Furthermore, more than one-third of respondents in this study claimed to have disturbed sleep about half the time or more after nocturnal awakening. The sleep disturbance was proportionately increased with the frequency of nocturnal voids and age. This finding is consistent with previous studies [5, 13, 14, 39]. As a result of sleep disturbance, nocturia has also been associated with excessive daytime sleepiness (7–18%), increased sick leaves (27–27%), insomnia (43%), psychiatric disorders (28–35%) and poorer health status (15–40%) [40, 41] In addition, people with nocturia have a lower productivity at work and decreased activity levels [6]. Consistently, the respondents with nocturia in this study also reported to experience fatigue, difficulty in concentrating, lacking of motivation to work, bad mood, forgetfulness and depression as consequences of disturbed sleep. Hence, it is obvious that nocturia should not be overlooked or underestimated due to its serious repercussions on sleep quality, work productivity and quality of life.

Treatment-seeking behaviour for LUTS is correlated with the symptom severity, degree of bother, costs and benefits of treatment [42, 43]. Consistently, this study found that those who had not sought treatment were presented with less than two nocturnal voids, less degree of sleep disturbance and lower bother score as compared to those who had sought treatment. To the best of our knowledge, this is the first study to report on the association between awareness of nocturia and treatment-seeking behaviour in Malaysia. Approximately two-thirds of respondents with nocturia in this study were not aware that nocturia is a medical condition and this was significantly associated with behaviour of not seeking medical attention. This finding suggests that poor awareness might be a factor affecting their treatment-seeking behaviour. In Taiwan, Chen and colleagues found that the lack of knowledge on nocturia appeared to be an important barrier to medical consultation among Taiwanese women [9]. In addition, a vast majority of those who had not sought treatment in the present study perceived that nocturia was not a significant medical issue to seek treatment. This appears consistent with finding by Low and colleagues who reported that majority of women in Northern Malaysia did not seek treatment for LUTS due to low level of disease awareness [11]. These findings could be attributed to poor knowledge pertaining to nocturia among Malaysians. As such, addressing the misconceptions about nocturia can help to increase the awareness, enhance treatment-seeking behaviour and also improve the overall quality of life among people with nocturia. Interestingly, this study revealed that most women did not seek treatment for nocturia as compared to men. This is in contrast with the finding from a study conducted in the primary care setting in Malaysia, which revealed the higher tendency for women to seek treatment when compared to men [44]. Further investigation is required to identify the sex difference in treatment-seeking behaviour among nocturia patients.

In the present study, convenience sampling was conducted instead of stratified sampling to define the population in Malaysia. Despite this limitation, the pool of subjects sampled were closely reflecting the real population in Malaysia. Although there was slightly difference between the sample and population in terms of age and sex, these differences were adjusted in the statistical analysis. Hence, the outcomes of this study are valuable to provide sights into prevalence of nocturia among Malaysians.

## Conclusions

The prevalence of nocturia among Malaysian adults is high and strongly influenced by age, sex, race and comorbidities. There is a significant correlation between frequency of nocturnal voids with sleep disturbance and degree of bother. However, the awareness among Malaysians pertaining to nocturia being a health issue was generally poor. This affects treatment-seeking behaviour as many are not aware that it is a symptom that requires medical attention. Contrary to common belief that it is a natural physiological symptom associated with ageing, nocturia as a condition deserves public attention, given its profound impact on quality of life that may be associated with increased morbidity and mortality. The findings from this study may serve as impetus to better address this issue via educational intervention.

## Supplementary Information

**Additional file 1**. Questionnaire for prevalence of nocturia among Malaysian adults - English version.

## Data Availability

The datasets used and/or analysed during the current study are available from the corresponding author on reasonable request. The English version of questionnaire is available in the supplementary information file.

## Abbreviations

ICS: International Continence Society
LUTS: Lower urinary tract symptoms
SD: Standard deviation
OR: Odd ratio
CI: Confidence interval
