# Supplementary material for: Prevalence of nocturia among community-dwelling adults: a population-based study in Malaysia
Source: BMC Urol. 2021 Jun 29;21:95. doi: 10.1186/s12894-021-00860-1 (PMC8243763; doi:10.1186/s12894-021-00860-1)
Supplement: Supplementary file 1 — Additional file 1. Questionnaire for prevalence of nocturia among Malaysian adults - English version. [file 12894_2021_860_MOESM1_ESM.pdf]

Ref. No:______________

Research Title: Prevalence of Nocturia* among Malaysian Adults: A population-based study

This questionnaire-based research study primarily focuses on the prevalence, disease patterns and level of awareness of nocturia among Malaysians. This study will serve as an important feedback to all stakeholders especially health authorities, healthcare professionals and the general public to better address this underreported disease.

Hence, we would like to welcome your kind participation in this study by spending 15 minutes to complete this questionnaire. Your participation is of great help to us.

NOTE: Please answer the questions honestly without referring to anyone or any available resources.

Nocturia is defined as the need to wake from sleep during the night to pass urine.

Part A : Demographic Information

1. Please provide your age as of 31 Dec 2019 :___________________

2. Sex
- □ Male
- □ Female

3. Race
- □ Malay
- □ Chinese
- □ Indian
- □ Others. Please specify : ______________________

4. Employment status
- □ Student
- □ Employed & receiving salary. Please state your occupation :___________
- □ Self-employed. Please state your occupation :___________
- □ Unemployed

5. Please provide your state of residence :___________________

Part B: Present medical condition

6. Do you have any of the following conditions? (Tick one or more boxes)

□ **High blood pressure**
□ **Diabetes**
□ **Kidney disease**
□ **Heart failure**
□ **Enlarged prostate**
□ **Overactive bladder**
□ **Prostate cancer**
□ **Bladder cancer**
□ **Snoring**
□ **Others. Please state** : ______________________________

7. Do you currently experience the following symptoms? (Tick one or more boxes)

□ **Post urination dribble (small amount of urine that leaks out right after urination)**

□ **Frequent urination during the day**

□ **Frequent urination at night**

□ **Frequent urinary urgency (sudden urge to urinate)**

□ **Urinary incontinence (inability to hold urine back leading to uncontrolled urination)**

□ **Slow urine flow**

□ **Feeling of incomplete bladder emptying after urination**

□ **Others. Please state** : ______________________________

8. Do you consume any of the following drinks in the late evening or before bedtime? (Tick one or more boxes)

□ **Large volume of water**
□ **Tea**
□ **Coffee**
□ **Alcohol**
□ **Others. Please state** : ______________________________
□ **None of the above**

Part C: Night urination and sleeping issues

9. **Do you think night time urination is a medical condition?**

   □ **Yes**
   □ **No**

10. **On average, how many times do you wake up from your sleep at night to urinate because of the urge to urinate?**

   □ **None**
   □ **Once**
   □ **Twice**
   □ **Three times**
   □ **Four times**
   □ **Five times or more**

If your answer is 'NONE' for Question 10, please proceed to Question 15.
If your answer is other than 'NONE' for Question 10, please proceed to Question 11.

11. **How often do you face difficulty in falling back asleep after waking up in the middle of the night?**

   □ **Almost always**
   □ **More than half the time**
   □ **About half the time**
   □ **Less than half the time**
   □ **Not at all**

12. **Do you experience the following conditions if you don't get enough sleep? (Tick one or more boxes)**

   □ **Fatigue**
   □ **Bad mood**
   □ **Difficulty in concentrating**
   □ **Depression**
   □ **Forgetfulness**
   □ **Lack of motivation to work**
   □ **Lack of sex drive**
   □ **If male; difficulty in getting an erection**
   □ **Others. Please state : ______________________________**

13. On a scale of 1 to 10, please indicate how bothersome night time urination is to you.

[Not bothersome at all] 1 – 2 – 3 – 4 – 5 – 6 – 7 – 8 – 9 – 10 [Extremely bothersome]

14. Have you sought medical attention for your night time urination?

□ Yes. Please choose one of the following:

- ❑ **I am happy with the treatment.**
- ❑ **The treatment only partially solves my problem.**
- ❑ **The treatment is not helping.**

□ No. Please choose one of the following:

- ❑ **But I will consider seeking treatment.**
- ❑ **I am shy to seek treatment.**
- ❑ **It is not a significant issue to seek treatment.**
- ❑ **Others. Please state : ____________________________**

15. Which medical personnel have you seen or would consider seeing to seek medical attention if you have night time urination? (Tick one or more boxes)

□ General practitioner (a doctor based in the community with no specialization)

□ Urologist (a doctor who specialises in urinary system)

□ Gynaecologist (a doctor who specialises in women's diseases)

□ Nephrologist (a doctor who specialises in kidney disorders)

□ Pharmacist
□ Others. Please state : ____________________________

Thank you for your participation!
